# Calcium-Mediated Induction of Paradoxical Growth following Caspofungin Treatment Is Associated with Calcineurin Activation and Phosphorylation in Aspergillus fumigatus

**DOI:** 10.1128/AAC.00263-15

**Published:** 2015-07-16

**Authors:** Praveen R. Juvvadi, Alberto Muñoz, Frédéric Lamoth, Erik J. Soderblom, M. Arthur Moseley, Nick D. Read, William J. Steinbach

**Affiliations:** a Division of Pediatric Infectious Diseases, Department of Pediatrics, Duke University Medical Center, Durham, North Carolina, USA; b Manchester Fungal Infection Group, Institute of Inflammation and Repair, University of Manchester, Manchester, United Kingdom; c Infectious Diseases Service, Department of Medicine, Lausanne University Hospital, Lausanne, Switzerland; d Institute of Microbiology, Lausanne University Hospital, Lausanne, Switzerland; e Duke Proteomics Facility, Institute for Genome Sciences and Policy, Duke University, Durham, North Carolina, USA; f Department of Molecular Genetics and Microbiology, Duke University Medical Center, Durham, North Carolina, USA

## Abstract

The echinocandin antifungal drug caspofungin at high concentrations reverses the growth inhibition of Aspergillus fumigatus, a phenomenon known as the “paradoxical effect,” which is not consistently observed with other echinocandins (micafungin and anidulafungin). Previous studies of A. fumigatus revealed the loss of the paradoxical effect following pharmacological or genetic inhibition of calcineurin, yet the underlying mechanism is poorly understood. Here, we utilized a codon-optimized bioluminescent Ca^2+^ reporter aequorin expression system in A. fumigatus and showed that caspofungin elicits a transient increase in cytosolic free Ca^2+^ ([Ca^2+^]_c_) in the fungus that acts as the initial trigger of the paradoxical effect by activating calmodulin-calcineurin signaling. While the increase in [Ca^2+^]_c_ was also observed upon treatment with micafungin, another echinocandin without the paradoxical effect, a higher [Ca^2+^]_c_ increase was noted with the paradoxical-growth concentration of caspofungin. Treatments with a Ca^2+^-selective chelator, BAPTA [1,2-bis(*o*-aminophenoxy)ethane-*N*,*N*,*N*′,*N*′-tetraacetic acid], or the L-type Ca^2+^ channel blocker verapamil abolished caspofungin-mediated paradoxical growth in both the wild-type and the echinocandin-resistant (EMFR-S678P) strains. Concomitant with increased [Ca^2+^]_c_ levels at higher concentrations of caspofungin, calmodulin and calcineurin gene expression was enhanced. Phosphoproteomic analysis revealed that calcineurin is activated through phosphorylation at its serine-proline-rich region (SPRR), a domain previously shown to be essential for regulation of hyphal growth, only at a paradoxical-growth concentration of caspofungin. Our results indicate that as opposed to micafungin, the increased [Ca^2+^]_c_ at high concentrations of caspofungin activates calmodulin-calcineurin signaling at both a transcriptional and a posttranslational level and ultimately leads to paradoxical fungal growth.

## INTRODUCTION

The echinocandin antifungal agent caspofungin is a β-1,3-glucan synthase inhibitor commonly used as second-line therapy for invasive aspergillosis (1). When used at high concentrations (>1 μg/ml) *in vitro*, caspofungin is known to reverse the expected growth inhibition of Aspergillus fumigatus in a phenomenon called the “paradoxical effect” (2). We have previously shown that pharmacologic inhibition of A. fumigatus calcineurin with FK506, deletion of the gene encoding the catalytic subunit of calcineurin (Δ*cnaA*), or deletion of the gene encoding the major transcription factor in the calcineurin pathway (Δ*crzA*) abolished the caspofungin-mediated paradoxical growth (3). Similar to increases in chitin content following treatment with high caspofungin concentrations in Candida albicans (4), we also noted a compensatory transcriptional upregulation of chitin synthases and increased chitin content following caspofungin treatment in A. fumigatus (5). However, the exact mechanism responsible for the calcineurin-mediated paradoxical reversal of growth inhibition at high caspofungin concentrations remains unknown.

Regulation of gene expression in response to Ca^2+^ signaling is one of the most explored functions of calcineurin. The critical target of calcineurin is the NFAT family of transcription factors during T-cell activation (6). In resting cells, NFAT proteins are phosphorylated and are retained in the cytoplasm. The fungal ortholog of NFAT, Crz1/CrzA, has also been shown to be phosphorylated (7, 8). Calcineurin is activated through binding of Ca^2+^/calmodulin (CaM) and then dephosphorylates the cytosolic form of Crz1/CrzA, which is then translocated to the nucleus for the activation of downstream targets (7, 9). Based on our previous results demonstrating the involvement of the calcineurin pathway in the paradoxical effect, we hypothesized that a mechanism for the paradoxical effect may involve a transient increase in cytosolic free Ca^2+^ ([Ca^2+^]_c_) in the fungal cell following treatment with high concentrations of caspofungin. This Ca^2+^ increase then results in the activation of calmodulin-calcineurin signaling, which in turn leads to growth recovery of the fungus through compensatory cell wall remodeling.

In the present study, we investigated the mechanism for paradoxical growth observed during treatment with higher concentrations of caspofungin by analyzing [Ca^2+^]_c_ changes and calcineurin activation following treatment of A. fumigatus with the two different echinocandin antifungals, caspofungin and micafungin.

## MATERIALS AND METHODS

### Strains, media, and growth conditions.

The wild-type A. fumigatus strain AF293, the A. fumigatus echinocandin-resistant strain EMFR-S678P, the A. fumigatus CEA10 strain, and the CEA10 (AEQ) strain expressing aequorin (A. Muñoz, M. Bertuzzi, J. Bettgenhäuser, N. Iakobachvili, E. M. Bignell, and N. D. Read, submitted for publication) were used for radial growth assays. The respective strains were cultured on glucose minimal medium (GMM) agar in the absence or presence of caspofungin (1 or 4 μg/ml) alone and in the presence of BAPTA [1,2-bis(*o*-aminophenoxy)ethane-*N*,*N*,*N*′,*N*′-tetraacetic acid] (1, 2, and 3 mM), CaCl_2_ (2.5 and 5 mM), verapamil (1 mM), or trifluoperazine (TFP; 10, 15, and 20 μM) to examine their effects on paradoxical growth. The growth assays were performed for 5 days at 37°C.

### Measurement of [Ca^2+^]_c_ concentrations in fungal cells.

A. fumigatus CEA10 (AEQ) expressing the bioluminescent Ca^2+^-sensitive reporter aequorin (Muñoz et al., submitted) was grown in liquid GMM in white 96-well microtiter plates containing 2.5 μM aequorin cofactor coelenterazine (Biosynth AG, Rietlistr, Switzerland) for 18 h at 28°C in the dark. Each treatment was repeated in six wells in each microtiter plate, and experiments were repeated at least three times. The luminescence was recorded using methods previously described (10) for a total period of 1 h while the fungus growing in each well was treated with either caspofungin or micafungin at 4 μg/ml. Cultures in each well were also pretreated for 30 min with 1 mM verapamil, 20 μM TFP, or 1 mM BAPTA before the addition of the antifungal drugs. The mathematical conversion of luminescence values (relative luminescence units [RLUs]) into cytosolic free calcium ([Ca^2+^]_c_) concentrations was carried out as described previously (10).

### Real-time reverse transcription-PCR (RT-PCR) analysis.

Expression of calmodulin- and calcineurin-encoding genes, *cmdA* and *cnaA*, respectively, was performed in the A. fumigatus (AF293) strain in the absence and presence of caspofungin. Conidia (10^6^/ml) were cultured in GMM broth under shaking conditions (200 rpm) for 20 h at 37°C. After 20 h, caspofungin (1 and 4 μg/ml) was added to the medium and cultures were incubated at 200 rpm for 4 h at 37°C. The resulting hyphae were harvested by vacuum filtration, washed extensively with cold sterile distilled water, and immediately frozen in liquid nitrogen. Total RNA was extracted using the RNeasy minikit (Qiagen) and treated with DNase I (Ambion). Total RNA (600 ng) was subjected to first-strand cDNA synthesis using the Tetro cDNA synthesis kit (Bioline). Real-time PCR assays were performed in triplicate using the iQ5 real-time PCR detection system (Bio-Rad) with 20-μl reaction volumes containing 2× Sensimix SYBR and fluorescein kit (Bioline), 0.2 μl of each primer, and 2 μl of a 1:5 dilution of the cDNA. The threshold cycle (2^−ΔΔ*CT*^) analytic method (11) normalized to beta-tubulin was used to calculate expression changes. Results are the means (± standard deviations) of results from three triplicate assays.

### Detection of calcineurin phosphorylation by liquid chromatography-tandem mass spectrometry (LC-MS/MS).

The A. fumigatus strain (AF293) expressing CnaA-enhanced green fluorescent protein (EGFP) was cultured in the presence of caspofungin (1 and 4 μg/ml) or micafungin (1 and 4 μg/ml) under shaking conditions (200 rpm) for 24 h at 37°C. The mycelia were harvested by washing with cold distilled water (200 ml) over a sintered glass funnel using a vacuum. The collected mycelia (0.5 to 0.75 mg [wet weight]) were immediately frozen in liquid nitrogen. Total cell extracts were obtained by grinding the mycelial tissue to fine powder using a mortar and pestle in liquid nitrogen and suspended in 5 ml lysis buffer (10 mM Tris-HCl, pH 7.5, 150 mM NaCl, 0.5 mM EDTA, 0.01% Triton X-100, 1 mM dithiothreitol [DTT], 1 mM phenylmethylsulfonyl fluoride [PMSF], 1:100 protease inhibitory cocktail). The homogenized mycelial extracts were first centrifuged at 5,000 rpm for 10 min at 4°C to remove cell debris. After this, the crude supernatant was again clarified by centrifugation at 7,000 rpm for 15 min at 4°C. Total protein in the crude extract was quantified by the Bradford method and normalized to contain ∼10 mg protein in a 5-ml volume of the sample before GFP-Trap affinity purification (Chromotek) using 35 μl of GFP-Trap agarose beads and processed for TiO_2_ phosphopeptide enrichment and mass spectrometry as previously described (12). The dried phosphopeptide enriched samples were resuspended in 10 μl of 2% acetonitrile, 0.1% formic acid, 10 mM citric acid and subjected to chromatographic separation on a Waters NanoAcquity ultraperformance liquid chromatograph (UPLC) equipped with a 1.7-μm BEH130 C_18_ 75-μm (inside diameter [i.d.]) by 250-mm reversed-phase column. The mobile phase consisted of (A) 0.1% formic acid in water and (B) 0.1% formic acid in acetonitrile. Following a 5-μl injection, peptides were trapped for 5 min on a 5-μm Symmetry C_18_ 180-μm (i.d.) by 20-mm column at 20 μl/min in 99.9% A. The analytical column was held at 5% B for 5 min and then switched in-line, and a linear elution gradient of 5% B to 40% B was performed over 90 min at 300 nl/min. The analytical column was connected to a fused silica PicoTip emitter (New Objective, Cambridge, MA) with a 10-μm tip orifice and coupled to an LTQ-Orbitrap XL mass spectrometer. In some experiments, the analytical column was connected to a fused silica PicoTip emitter (New Objective, Cambridge, MA) with a 10-μm tip orifice and was coupled to a Waters Synapt G2 quadrupole time of flight (QTOF) mass spectrometer through an electrospray interface operating in a data-dependent mode of acquisition. The instrument was set to acquire a precursor MS scan in the Orbitrap spectrometer from *m/z* 400 to 2,000 with *r* = 60,000 at *m/z* 400 and a target automatic gain control (AGC) setting of 1e6 ions. In a data-dependent mode of acquisition, MS/MS spectra of the three most abundant precursor ions were acquired in the Orbitrap spectrometer with *r* = 7,500 at *m/z* with a target AGC setting of 2e5 ions. Maximum fill times were set to 1,000 ms for full MS scans and 500 ms for MS/MS scans with minimum MS/MS triggering thresholds of 5,000 counts. For all experiments, fragmentation occurred in the LTQ linear ion trap with a collision-induced dissociation (CID) energy setting of 35% and a dynamic exclusion of 60 s was employed for previously fragmented precursor ions. When using the Waters Synapt G2 QTOF mass spectrometer through an electrospray interface operating in a data-dependent mode of acquisition, the instrument was set to acquire a precursor MS scan from *m/z* 50 to 2,000 with MS/MS spectra acquired for the three most abundant precursor ions. For all experiments, charge-dependent CID energy settings were employed and a 120-s dynamic exclusion was employed for previously fragmented precursor ions.

## RESULTS

### Calcium is required for the induction of paradoxical growth due to caspofungin.

**FIG 1:**
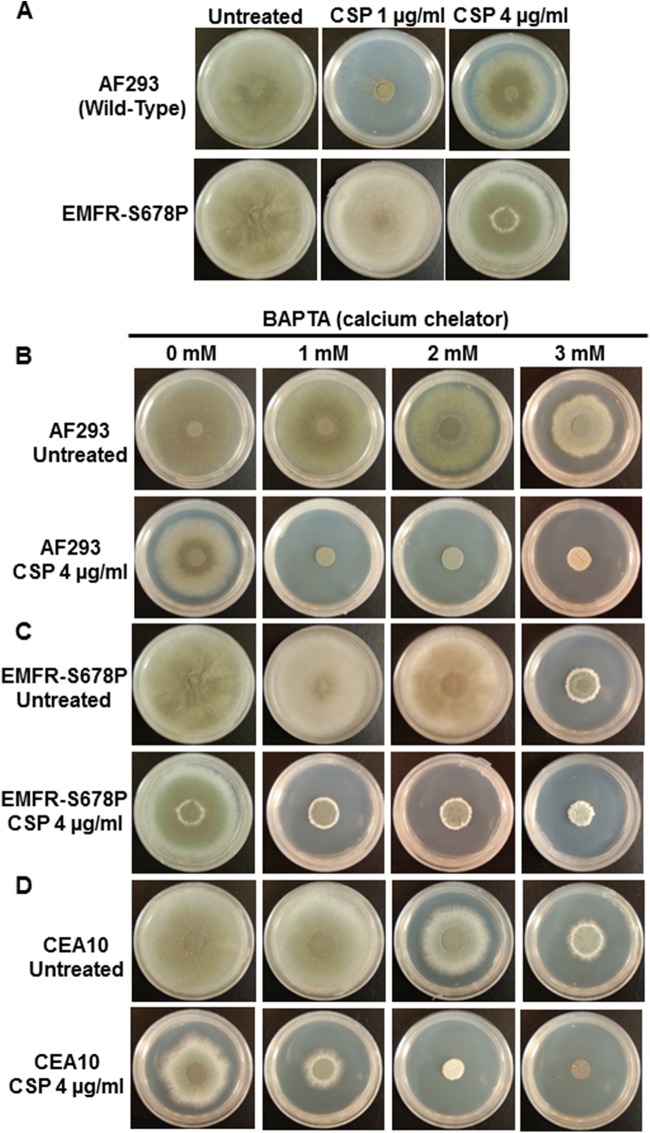
(A) Paradoxical growth observed with caspofungin (CSP) in the AF293 strain but not in the echinocandin-resistant strain (EMFR-S678P). Caspofungin at 1 μg/ml is active against the AF293 strain, causing a significant growth defect, but higher concentrations (2 and 4 μg/ml) result in a paradoxical growth recovery. The EMFR-S678P strain demonstrated resistance to caspofungin at all concentrations. (B to D) The Ca^2+^ chelator BAPTA blocked caspofungin-induced paradoxical growth of AF293 and CEA10 and also effectively abrogated echinocandin resistance of the EMFR-S678P strain. In all cases, 1 × 10^4^ spores of each strain were spotted onto GMM agar plates and cultured for 5 days at 37°C.

While the echinocandins (micafungin and caspofungin) inhibit the growth of the wild-type Aspergillus fumigatus at lower concentrations (1 μg/ml), only caspofungin at high concentrations (>1 μg/ml) induces paradoxical growth (Fig. 1A). As shown in Fig. 1A, in contrast to the wild-type A. fumigatus AF293 strain, the EMFR-S678P strain is resistant to caspofungin due to a point mutation in the *fksA* gene encoding the β-1,3-glucan synthase (13). To investigate if Ca^2+^-dependent events are the primary cause for triggering the paradoxical growth response at higher concentrations of caspofungin, we first determined if extracellular Ca^2+^ is required for paradoxical growth. The wild-type A. fumigatus AF293 strain was grown in the presence of various concentrations of the Ca^2+^-selective chelator BAPTA alone or in combination with a higher concentration (4 μg/ml) of caspofungin (Fig. 1B). Although BAPTA alone at higher concentrations (>2 mM) had a growth-inhibitory effect, 1 mM BAPTA alone did not have any basal growth-inhibitory effect. However, paradoxical growth due to caspofungin at 4 μg/ml was abolished by 1 mM BAPTA, indicating the requirement of extracellular Ca^2+^ for paradoxical growth. We next examined if chelating extracellular Ca^2+^ could also induce growth inhibition in the echinocandin-resistant EMFR-S678P strain (Fig. 1B) (13). Chelation of the extracellular Ca^2+^ produced a similar response in this strain in the presence of 4 μg/ml of caspofungin (Fig. 1C). To further verify if the inhibition of the paradoxical response by BAPTA is strain independent, we next tested the effect of BAPTA in combination with caspofungin in another clinical isolate, CEA10 (Fig. 1D). Growth of the CEA10 strain, which also showed the paradoxical response with 4 μg/ml caspofungin, was also inhibited in the presence of 1 mM BAPTA. Taken together, these results revealed the requirement of extracellular Ca^2+^ for the paradoxical growth response. To confirm these results, we treated the strains with the less selective Ca^2+^ chelator EGTA and found that 4 mM EGTA completely blocked paradoxical growth, showing the importance of extracellular Ca^2+^ for the regulation of growth following caspofungin treatment (see Fig. S1 in the supplemental material).

**FIG 2:**
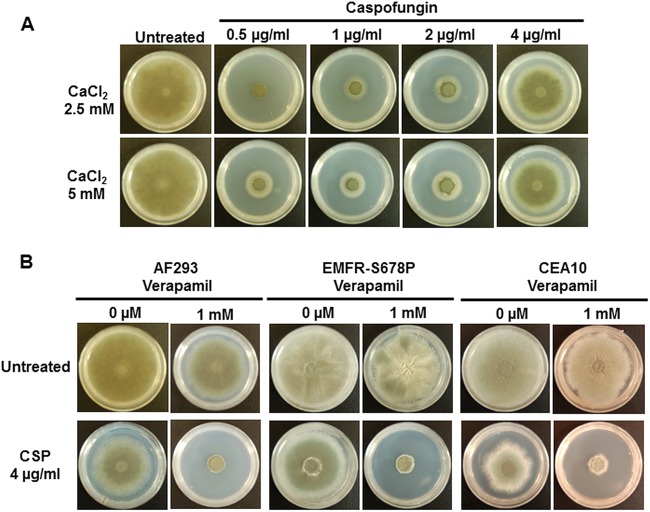
(A) Inclusion of CaCl_2_ slightly rescued growth inhibition by caspofungin at 0.5 to 2 μg/ml. Spores (1 × 10^4^) of the AF293 strain were cultured in the presence of various concentrations of caspofungin supplemented with 2.5 mM and 5 mM CaCl_2_. Growth was monitored for a period of 5 days at 37°C. (B) The Ca^2+^ channel blocker verapamil at 1 mM inhibited caspofungin (CSP) paradoxical growth, indicating the requirement of extracellular Ca^2+^ for inducing paradoxical growth. In all cases, 1 × 10^4^ spores of each strain were spotted onto GMM agar in the absence or presence of verapamil in combination with 4 μg/ml caspofungin for 5 days at 37°C.

In order to more clearly understand the link between Ca^2+^ and paradoxical growth, we next examined the effect of external Ca^2+^ at growth-inhibitory concentrations of caspofungin (0.5 to 2 μg/ml). As shown in Fig. 2A, the inclusion of 2.5 mM and 5 mM CaCl_2_ resulted in only a slight growth recovery in comparison to the lack of exogenous CaCl_2_ (Fig. 1B). However, the requirement of Ca^2+^ for paradoxical growth was further confirmed by addition of the L-type Ca^2+^ channel blocker verapamil (14, 15), which completely abolished paradoxical growth when used at a concentration of 1 mM in all strains, including the EMFR-S678P echinocandin-resistant strain (Fig. 2B).

### Caspofungin treatment causes an increase in cytosolic Ca^2+^ levels.

**FIG 3:**
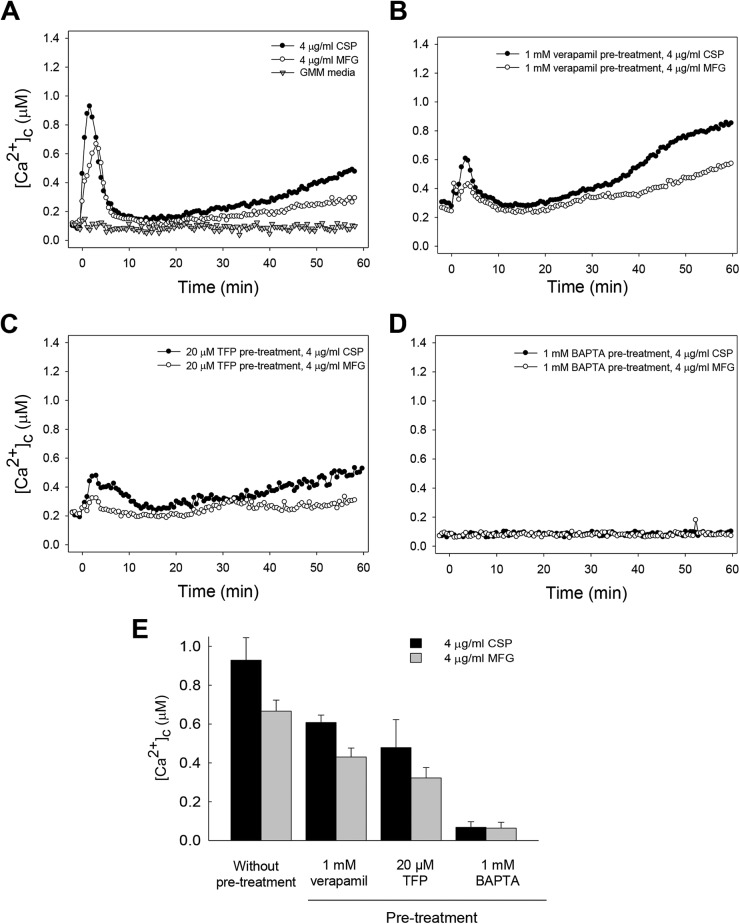
Caspofungin (CSP) and micafungin (MFG) induced perturbation in cytosolic free calcium ([Ca^2+^]_c_) concentration with reproducible Ca^2+^ signatures. Four micrograms per milliliter of each echinocandin or GMM alone was added, and the [Ca^2+^]_c_ was monitored over 60 min. (A to D) A. fumigatus CEA10 (AEQ) grown in the wells of multiwell plates was either treated directly with caspofungin or micafungin (A), pretreated for 30 min with 1 mM verapamil (B), pretreated with 20 μM trifluoperazine (TFP) (C), or pretreated with 1 mM BAPTA (D) before the addition of 4 μg/ml of the drugs. (E) Summary of the mean maximum [Ca^2+^]_c_ amplitudes for each of the treatments ∼3 min after the drug treatments.

To quantify changes in cytosolic free ([Ca^2+^]_c_) levels, an A. fumigatus strain expressing the codon-optimized aequorin in a CEA10 background was used with the aequorin under the control of the constitutive *gpdA* promoter (Muñoz et al., submitted). The A. fumigatus CEA10 (AEQ) strain also showed the paradoxical-growth response, which was inhibited by the presence of BAPTA and verapamil (see Fig. S2A and B in the supplemental material), similar to that of the untransformed CEA10 strain (Fig. 1D and 2B). The CEA10 (AEQ) strain was incubated in the dark for 18 h at 28°C and then treated with 4 μg/ml caspofungin, and [Ca^2+^]_c_ was measured over a period of 60 min (Fig. 3A). Following the addition of 4 μg/ml caspofungin, there was a significant increase in [Ca^2+^]_c_ up to ∼0.9 μM followed by a pronounced decrease in [Ca^2+^]_c_ to just above resting level, which after ∼15 min then steadily increased until the end of the experiment.

In order to determine whether [Ca^2+^]_c_ changes were specific to caspofungin, we also measured the influence on [Ca^2+^]_c_ of another echinocandin, micafungin, which does not exhibit paradoxical growth at higher concentrations. The results with 4 μg/ml of micafungin showed a similar trend as with caspofungin, but the changes in [Ca^2+^]_c_ were consistently and significantly lower (Fig. 3A).

The initial large transient [Ca^2+^]_c_ increases in response to the two echinocandins were greatly inhibited after pretreatment for 30 min with the Ca^2+^ channel blocker verapamil when used at the low concentration of 1 mM (Fig. 3B), although the [Ca^2+^]_c_ resting level was generally increased, as previously reported for Neurospora crassa (16). A 30-min pretreatment with the calmodulin inhibitor trifluoperazine (TFP) at 20 μM also significantly reduced the amplitude of the [Ca^2+^]_c_ response to 4 μg/ml of either echinocandin (Fig. 3C).

To further confirm the requirement of extracellular Ca^2+^ for paradoxical growth, [Ca^2+^]_c_ levels were monitored following a similar pretreatment with 1 mM BAPTA. As shown in Fig. 3D, there was no change in the resting level of [Ca^2+^]_c_. The results with 4 μg/ml of micafungin alone or in the presence of inhibitors also showed an increase in the [Ca^2+^]_c_ amplitude. However, the transient changes in [Ca^2+^]_c_ and the overall Ca^2+^ signatures obtained with micafungin were consistently lower and different in all the cases from those obtained with caspofungin (Fig. 3E).

### Paradoxical growth is associated with the activation of the calmodulin and calcineurin pathway.

**FIG 4:**
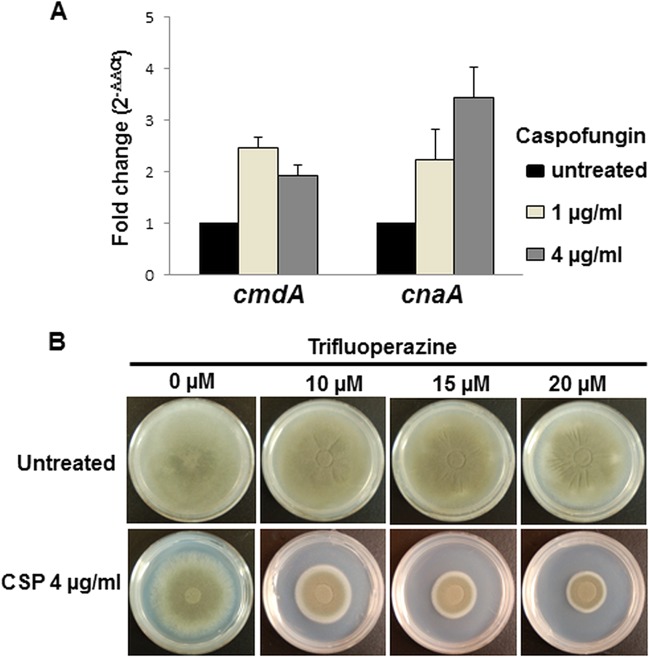
(A) Quantification of the transcription of calmodulin (*cmdA*) and calcineurin A (*cnaA*) by real-time reverse transcription-PCR in the AF293 strain following exposure to increasing concentrations of caspofungin. Conidia were grown for 20 h in liquid GMM in the absence of any drug. Caspofungin was added at concentrations of 0, 1, and 4 μg/ml for an additional 4-h incubation. Results are presented as the mean fold change (2^−ΔΔ*CT*^) ± standard deviation compared to the untreated condition (black columns). (B) The calmodulin inhibitor trifluoperazine inhibited caspofungin (CSP) paradoxical growth with 4 μg/ml caspofungin in a dose-dependent manner, consistent with the caspofungin-induced activation of calmodulin being necessary to activate downstream calcineurin signaling. Spores (1 × 10^4^) of the AF293 strain were cultured in the presence of various concentrations of TFP alone or in combination with 4 μg/ml caspofungin. Growth was monitored for a period of 5 days at 37°C.

To further investigate the link between the increase in [Ca^2+^]_c_ levels in the fungal cells and the primary downstream signaling cascade regulating growth in response to caspofungin, we analyzed the expression profile of key genes, calmodulin (*cmdA*) and calcineurin A (*cnaA*), following treatment with 1 and 4 μg/ml of caspofungin. Consistent with the radial growth assays, caspofungin treatment resulted in increased expression of both *cmdA* and *cnaA* genes (Fig. 4A). There was a 3.5-fold increase in expression of *cnaA* in the presence of 4 μg/ml of caspofungin. Because the anticalmodulin drug TFP reduced the [Ca^2+^]_c_ increase in response to caspofungin, its effect on paradoxical growth was also tested. As shown in Fig. 4B, 20 μM TFP inhibited paradoxical growth, which is consistent with calmodulin activation being required for the paradoxical growth response by activating calcineurin. These collective results provide strong evidence that increased expression of both calmodulin and calcineurin, together with their activation in response to the increase in [Ca^2+^]_c_ following caspofungin treatment, is important for the induction of paradoxical growth.

### Calcineurin is differentially phosphorylated following caspofungin and micafungin treatment.

**FIG 5:**
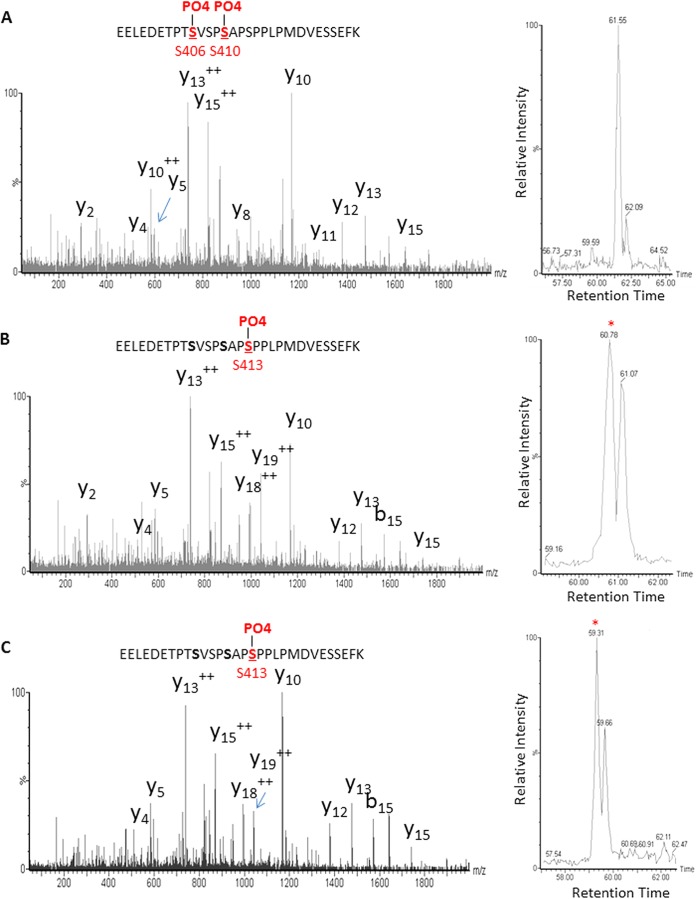
Phosphorylation profile of the A. fumigatus CnaA SPRR in the presence of caspofungin. (A and B) Tandem mass spectra of EELEDETPT[pS]VSP[pS]APSPPLPMDVESSEFK (A) and EELEDETPTSVSPSAP[pS]PPLPMDVESSEFK (B) in the CnaA subunit reveal three unique phosphorylated serine residues (S406, S410, and S413) clustered within the SPRR following treatment with 4 μg/ml caspofungin. The presence of each identified C-terminal (y) and N-terminal (b) product ion is indicated within the peptide sequence. The y-series and b-series ions are C-terminal and N-terminal ions, respectively, and qualitatively describe the confidence in identification from the MS/MS spectrum. For additional verification of the unique localization of phosphorylation between the two peptides, corresponding full MS extracted ion chromatograms of *m/z* 1,131.14 (±10 ppm) and 1,104.48 (±10 ppm) are shown on the right of each mass spectrum and illustrate a clear chromatographic shift in retention time between the species. (C) Phosphorylation profile of the A. fumigatus CnaA SPRR in the presence of micafungin. Tandem mass spectra of the phosphorylated peptide EELEDETPTSVSPSAP[pS]PPLPMDVESSEFK in the CnaA subunit reveal a unique phosphorylated serine residue (S413) within the SPRR. The presence of each identified C-terminal (y) and N-terminal (b) product ion is indicated within the peptide sequence. For additional verification of the unique localization of phosphorylation, the corresponding full MS extracted ion chromatogram of *m/z* 1,104.48 (±10 ppm) is shown on the right of the mass spectrum and illustrates a clear chromatographic shift in retention time between the species.

Our earlier studies revealed that CnaA is activated through phosphorylation at a unique serine-proline-rich region (SPRR) (17). To verify if calcineurin activation through phosphorylation is also required for caspofungin-mediated paradoxical growth, we next analyzed the phosphorylation status of CnaA after treatment with caspofungin at 1- and 4-μg/ml concentrations (Table 1). In order to purify CnaA for the phosphoproteomic analysis, the strains were grown in liquid GMM as shaking cultures for 24 h in the absence/presence of the respective echinocandins. Paradoxical growth was observed in shaking cultures at 24 h (data not shown). At growth-inhibitory concentrations of caspofungin (1 μg/ml), only a single serine residue (S537) was phosphorylated in the C terminus of CnaA and no serines in the SPRR were phosphorylated. However, at paradoxical-growth caspofungin concentrations (4 μg/ml), three serines (S406, S410, and S413) were phosphorylated in the SPRR (Table 1; Fig. 5A and B). Additionally, the CnaB regulatory subunit was phosphorylated at serine 21 (S21) in the N terminus (Table 1). To investigate the importance of increased phosphorylation of CnaA at the SPRR for supporting paradoxical growth at higher concentrations of caspofungin, a similar phosphoproteomic analysis was performed after treatment with micafungin at 1-μg/ml and 4-μg/ml concentrations. As shown in Table 1, only the C-terminal serine residue (S537) was phosphorylated on CnaA at 1 μg/ml of micafungin. In the presence of 4 μg/ml micafungin, only a single serine residue (S413) within the SPRR (Fig. 5C) along with the single serine residue (S537) in the C terminus was phosphorylated (Table 1). In all treatments, the phosphorylation of CnaB did not show any variation, indicating that the specific phosphorylation of serine residues (S406 and S410) in the CnaA SPRR may regulate the activation of the calcineurin complex. We postulate that through differential phosphorylation, calcineurin may exert transcriptional activation via nuclear translocation of CrzA to regulate paradoxical growth in response to caspofungin.

**TABLE 1:** Differential phosphorylation of CnaA and CnaB in response to caspofungin and micafungin treatment identified by TiO_2_ enrichment and LC-MS/MS analysis^*e*^

Condition/protein^*a*^	Peptide sequence^*b*^	Phosphorylated residue(s)	*m/z*	Charge	Mass error (ppm)	Mascot ion score^*c*^	Ascore localization probability^*d*^ (%)
Caspofungin 1							
CnaA	RI[pS]MSAGSGR	S537	551.25	2	−0.1	56.8	98
CnaB	A[pS]VGTSQLLDNIVSASNFDRDEVDR	S21	930.10	3	−2.9	76.1	99
Caspofungin 4							
CnaA	EELEDETPT[pS]VSP[pS]APSPPLPMDVESSEFK	S406, S410	1,131.14	3	−1.8	49.8	95, 99
CnaA	EELEDETPTSVSPSAP[pS]PPLPMDVESSEFK	S413	1,104.48	3	−1.2	64.9	95
CnaA	RI[pS]MSAGSGR	S537	551.25	2	1.2	50.5	97
CnaB	A[pS]VGTSQLLDNIVSASNFDRDEVDR	S21	930.1	3	−3.2	93.3	99
Micafungin 1							
CnaA	RI[pS]MSAGSGR	S537	551.25	2	0.8	54	88
CnaB	A[pS]VGTSQLLDNIVSASNFDRDEVDR	S21	930.1	3	−2.6	87	99
Micafungin 4							
CnaA	EELEDETPTSVSPSAP[pS]PPLPMDVESSEFK	S413	1,104.48	3	−3.9	65.2	99
CnaA	RI[pS]MSAGSGR	S537	551.25	2	0.2	69.7	94
CnaB	A[pS]VGTSQLLDNIVSASNFDRDEVDR	S21	930.10	3	−0.1	88.2	99

a Caspofungin 1 and caspofungin 4 indicate caspofungin used at 1 μg/ml and 4 μg/ml; micafungin 1 and micafungin 4 indicate micafungin used at 1 μg/ml and 4 μg/ml.

b [pS] indicates phosphorylated residue.

c Mascot identity score of >41 indicates identity or extensive homology (*P* < 0.05).

d Probability of phosphorylated residue localization based on Ascore algorithm.

e CnaA and CnaB subunit peptides contained uniquely identified phosphorylation residues or combinations of phosphorylation residues following treatment with caspofungin (1 and 4 μg/ml) and micafungin (1 and 4 μg/ml). Due to the proximity of multiple phosphorylatable residues within these peptides, mass spectra of each identification were submitted to an independent algorithm (Ascore) which assigns confidences to the localization of each phosphorylation.

## DISCUSSION

Although our previous studies have indicated the requirement of calcineurin and its downstream transcription factor, CrzA, for the caspofungin-mediated paradoxical growth response (3), the exact mechanism of how this process is regulated by calcineurin remains unknown. Considering the importance of Ca^2+^ signaling in the calcineurin pathway, we first investigated if extracellular Ca^2+^ is required for the paradoxical growth response by utilizing Ca^2+^-selective inhibitors. Paradoxical growth in response to high concentrations of caspofungin was abolished in the presence of Ca^2+^-selective inhibitors in both the wild-type and the echinocandin-resistant strain (EMFR-S678P). This suggested that inhibition of the Ca^2+^ signaling pathway may provide an alternative strategy to counteract caspofungin resistance in A. fumigatus.

Interestingly, although the paradoxical growth could be blocked by the Ca^2+^-selective inhibitors, inclusion of Ca^2+^ in the growth medium did not cause growth recovery in the presence of growth-inhibitory concentrations of caspofungin. This suggested that although Ca^2+^ could be an initial trigger to regulate paradoxical growth, only exogenous addition of Ca^2+^ may not be sufficient to activate the downstream signal transduction mechanisms regulating paradoxical growth. In order to verify and quantitate changes in free cytosolic calcium ([Ca^2+^]_c_) levels during the caspofungin treatment that promotes paradoxical growth, we utilized the codon-optimized bioluminescent Ca^2+^ reporter aequorin. This method was previously developed for 96-well plate luminometry to measure [Ca^2+^]_c_ dynamics in living populations of spore germlings or hyphae of Aspergillus and Neurospora (16, 18). [Ca^2+^]_c_ measurements in the presence of the two echinocandins revealed a significant increase with caspofungin, and the amplitude of this [Ca^2+^]_c_ response was inhibited by the calmodulin inhibitor TFP. It is possible that TFP inhibits the Ca^2+^ uptake, as the levels of [Ca^2+^]_c_ measured after TFP pretreatment for 30 min were reduced significantly. Previously, TFP was shown to block extracellular Ca^2+^ uptake in rat pituitary tumor cells (19) and inhibit [Ca^2+^]_c_ in human neutrophils by interfering with Ca^2+^-ATPase activity (20). Furthermore, supporting these observations was the increased expression of calmodulin- and calcineurin-encoding genes, *cmdA* and *cnaA*, respectively, in response to the increase in [Ca^2+^]_c_ following caspofungin treatment, revealing their importance for the induction of paradoxical growth.

As it is possible that *cnaA* expression and activation are responsible for the paradoxical growth, we next analyzed the phosphorylation status of both CnaA and CnaB in the presence of the two echinocandins (caspofungin and micafungin) to more clearly understand the mechanistic basis for caspofungin paradoxical growth at a posttranscriptional level. While the phosphorylation of CnaB at the serine 21 (S21) position remained unaltered at all the concentrations of echinocandins tested, increased phosphorylation of CnaA was noted in the unique serine-proline-rich region (SPRR) at positions S406, S410, and S413 only at paradoxical growth concentrations. We recently showed that phosphorylation of CnaA at a unique serine-proline-rich region is required for proper hyphal growth in A. fumigatus and that mutation of all of the 4 phosphorylated serine residues (S406, S408, S410, and S413) within the SPRR abolished the paradoxical growth due to caspofungin (17).

One caveat of this current study is that although paradoxical growth is achieved after a 3-day growth period on agar medium, the [Ca^2+^]_c_ measurements were performed in 18- to 20-h cultures due to limitations of the aequorin assays. Nonetheless, a clear effect on Ca^2+^ homeostasis was seen during these early stages of fungal colony formation. Disruption of fungal Ca^2+^ homeostasis by different antifungal proteins, peptides, and other drugs has been described previously (10, 14, 16, 21, 22). However, this is the first report of a clear link between the involvement of [Ca^2+^]_c_ and a downstream effect of an antifungal drug (the paradoxical effect). Specifically, after treatment with growth-inhibitory concentrations of caspofungin, only carboxy-terminal phosphorylation of calcineurin was noted, but following treatment with paradoxical-growth concentrations of caspofungin, the CnaA SPRR was phosphorylated at S406, S410, and S413 sites previously shown to be involved in the activation of calcineurin (17). Calmodulin and calcineurin form a regulated signaling network to direct paradoxical growth, and any disturbance in this pathway can cause inhibition of paradoxical growth. Nevertheless, we cannot rule out the possibility of additional compensatory mechanisms operating during paradoxical growth or other off-target effects of the two echinocandins. Based on the results obtained, Ca^2+^-calmodulin-calcineurin signaling is one primary mechanism involved in the regulation of paradoxical growth. This study also reveals that extracellular Ca^2+^ chelation can be a useful alternative to overcome echinocandin resistance. However, currently available Ca^2+^-modulating drugs might not be ideal due to potential clinical toxicity, so safer Ca^2+^ modulators could be used in combination with the echinocandins. Further work is also required to clearly understand how the dynamic change in the phosphorylation status of CnaA following treatment with the two echinocandins influences its interaction with downstream targets.

## Supplementary Material

Supplemental material
