# Supplementary material for: Calcium-Mediated Induction of Paradoxical Growth following Caspofungin Treatment Is Associated with Calcineurin Activation and Phosphorylation in Aspergillus fumigatus
Source: Antimicrob Agents Chemother. 2015 Jul 16;59(8):4946–55. doi: 10.1128/AAC.00263-15 (PMC4505252; doi:10.1128/AAC.00263-15)
Supplement: Supplemental material [file supp_59_8_4946__index.html]

Calcium-Mediated Induction of Paradoxical Growth following Caspofungin Treatment Is Associated with Calcineurin Activation and Phosphorylation in Aspergillus fumigatus — Supplemental material 

# Calcium-Mediated Induction of Paradoxical Growth following Caspofungin Treatment Is Associated with Calcineurin Activation and Phosphorylation in Aspergillus fumigatus

## Supplemental material

- Supplemental file 1 -

  Fig. S1 and S2

  PDF, 273K
