# Supplementary material for: Calcium-Mediated Induction of Paradoxical Growth following Caspofungin Treatment Is Associated with Calcineurin Activation and Phosphorylation in Aspergillus fumigatus
Source: Antimicrob Agents Chemother. 2015 Jul 16;59(8):4946–55. doi: 10.1128/AAC.00263-15 (PMC4505252; doi:10.1128/AAC.00263-15)
Supplement: Supplemental material [file AAC.00263-15_zac008154257so1.pdf]

Supplementary Figure S1

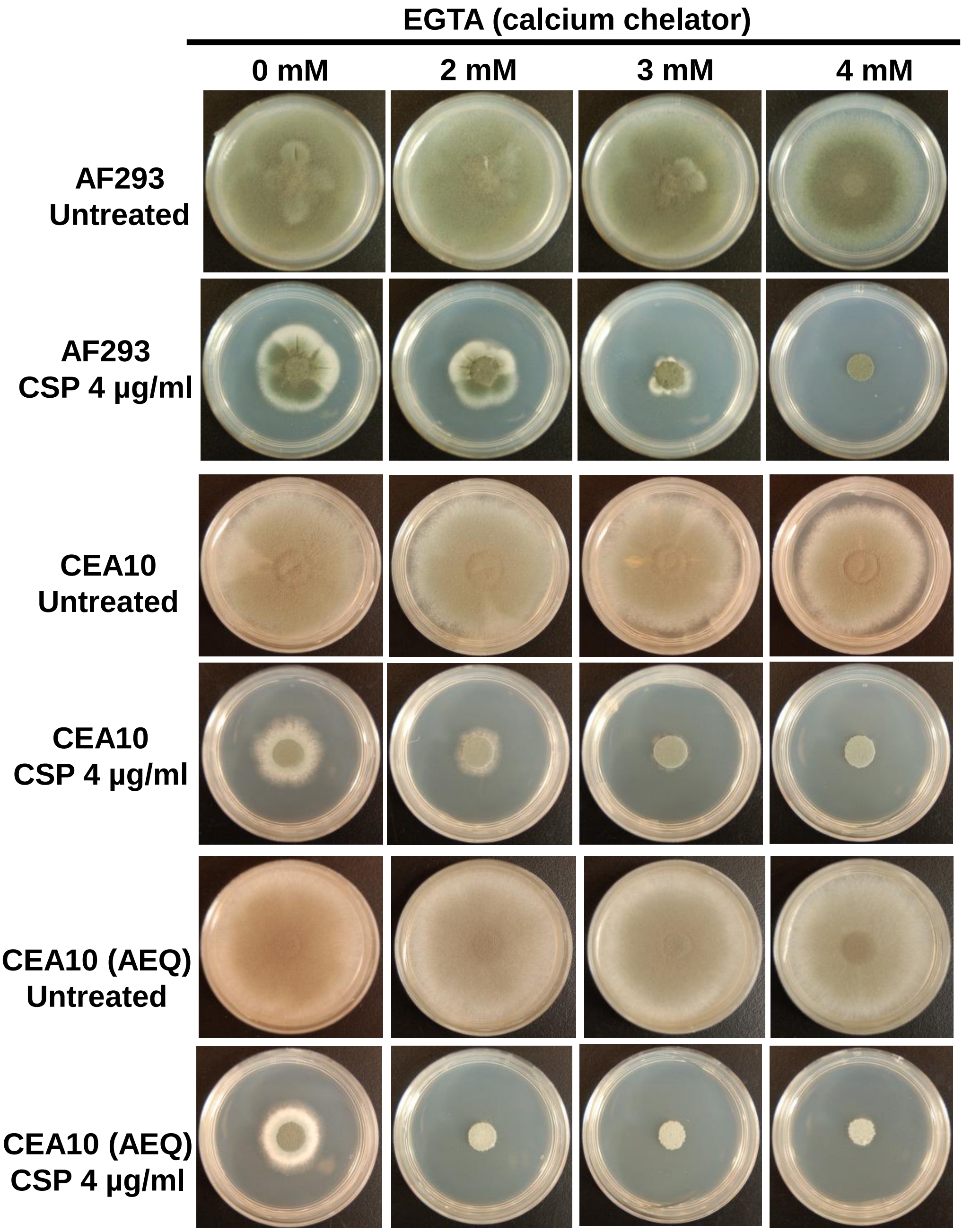

Figure S1. The respective strains (1 x 10^4 spores each) were spotted on GMM agar in the absence or presence of caspofungin (CSP) supplemented with increasing concentrations of the divalent cation chelator, EGTA, and cultured for 5 days at 37°C. Note the inhibitory effect of 4 mM EGTA on paradoxical growth induced by 4 µg/ml caspofungin.

Supplementary Figure S2

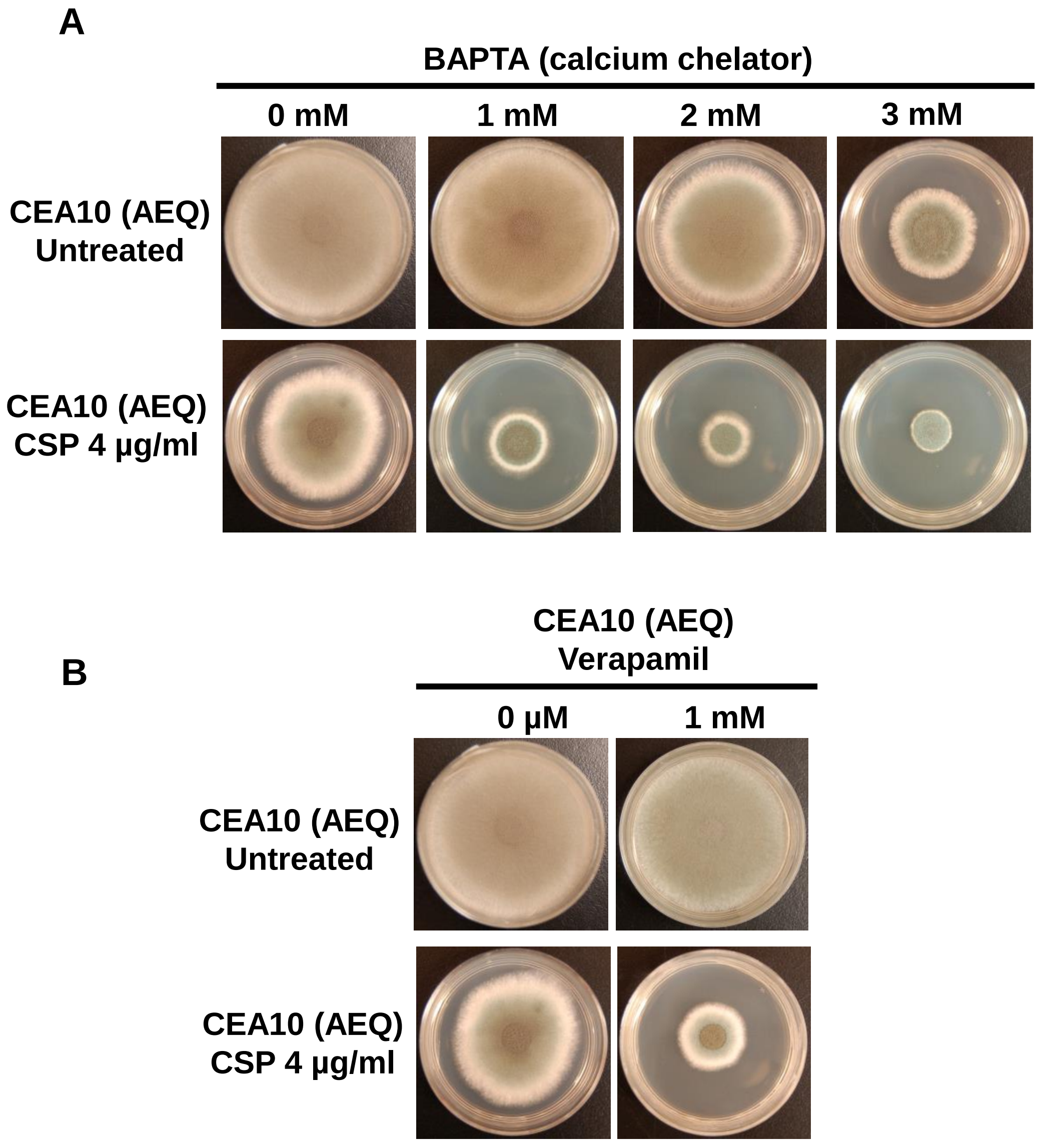

Figure S2. (A) The CEA10 (AEQ) strain expressing aequorin (1 x 10^4 spores) was spotted on GMM agar in the absence or presence of caspofungin (CSP) supplemented with increasing concentrations of the calcium chelator, BAPTA, and cultured for 5 days at 37°C. **(B)** The CEA10 (AEQ) strain was cultured in the absence or presence of caspofungin and 1 mM verapamil. The Ca^{2+}-channel blocker inhibited caspofungin-induced paradoxical growth.
